# Predicting potential lncRNA biomarkers for lung cancer and neuroblastoma based on an ensemble of a deep neural network and LightGBM

**DOI:** 10.3389/fgene.2023.1238095

**Published:** 2023-08-16

**Authors:** Zhenguo Su, Huihui Lu, Yan Wu, Zejun Li, Lian Duan

**Affiliations:** ^1^ Clinical Lab, Yantai Affiliated Hospital of Binzhou Medical University, Yantai, China; ^2^ Department of Thoracic Cardiovascular Surgery, Hunan Province Directly Affiliated TCM Hospital, Zhuzhou, China; ^3^ Geneis (Beijing) Co., Ltd., Beijing, China; ^4^ School of Computer Science, Hunan Institute of Technology, Hengyang, China; ^5^ Faculty of Pediatrics, The Chinese PLA General Hospital, Beijing, China; ^6^ Department of Pediatric Surgery, The Seventh Medical Center of PLA General Hospital, Beijing, China; ^7^ National Engineering Laboratory for Birth Defects Prevention and Control of Key Technology, Beijing, China; ^8^ Beijing Key Laboratory of Pediatric Organ Failure, Beijing, China

**Keywords:** lncRNA, biomarker, lung cancer, neuroblastoma, deep neural network, LightGBM

## Abstract

**Introduction:** Lung cancer is one of the most frequent neoplasms worldwide with approximately 2.2 million new cases and 1.8 million deaths each year. The expression levels of programmed death ligand-1 (PDL1) demonstrate a complex association with lung cancer. Neuroblastoma is a high-risk malignant tumor and is mainly involved in childhood patients. Identification of new biomarkers for these two diseases can significantly promote their diagnosis and therapy. However, *in vivo* experiments to discover potential biomarkers are costly and laborious. Consequently, artificial intelligence technologies, especially machine learning methods, provide a powerful avenue to find new biomarkers for various diseases.

**Methods:** We developed a machine learning-based method named LDAenDL to detect potential long noncoding RNA (lncRNA) biomarkers for lung cancer and neuroblastoma using an ensemble of a deep neural network and LightGBM. LDAenDL first computes the Gaussian kernel similarity and functional similarity of lncRNAs and the Gaussian kernel similarity and semantic similarity of diseases to obtain their similar networks. Next, LDAenDL combines a graph convolutional network, graph attention network, and convolutional neural network to learn the biological features of the lncRNAs and diseases based on their similarity networks. Third, these features are concatenated and fed to an ensemble model composed of a deep neural network and LightGBM to find new lncRNA–disease associations (LDAs). Finally, the proposed LDAenDL method is applied to identify possible lncRNA biomarkers associated with lung cancer and neuroblastoma.

**Results:** The experimental results show that LDAenDL computed the best AUCs of 0.8701, 107 0.8953, and 0.9110 under cross-validation on lncRNAs, diseases, and lncRNA‐disease pairs on Dataset 1, respectively, and 0.9490, 0.9157, and 0.9708 on Dataset 2, respectively. Furthermore, AUPRs of 0.8903, 0.9061, and 0.9166 under three cross‐validations were obtained on Dataset 1, and 0.9582, 0.9122, and 0.9743 on Dataset 2. The results demonstrate that LDAenDL significantly outperformed the other four classical LDA prediction methods (i.e., SDLDA, LDNFSGB, IPCAF, and LDASR). Case studies demonstrate that CCDC26 and IFNG-AS1 may be new biomarkers of lung cancer, SNHG3 may associate with PDL1 for lung cancer, and HOTAIR and BDNF-AS may be potential biomarkers of neuroblastoma.

**Conclusion:** We hope that the proposed LDAenDL method can help the development of targeted therapies for these two diseases.

## 1 Introduction

Long non-coding RNAs (lncRNAs) are non-coding RNAs with more than 200 nucleotides (Bertone et al., 2004; Peng et al., 2022a; Peng et al., 2022b). LncRNAs play an important role in the development and progression of various diseases (Lanjanian et al., 2021; Meng et al., 2021; Yang and Li 2021; Peng et al., 2022c). LncRNAs have dense associations with many diseases, for example, lung cancer, colorectal cancer, prostate cancer, and Alzheimer’s disease (Klattenhoff et al., 2013; Tan et al., 2013; Chakravarty et al., 2014; He et al., 2014; Zhang et al., 2014). LncRNA H19 is associated with the under-regulation of renal carcinoma cells (Wang et al., 2015). The expression of EGOT in breast cancer is much lower than one in adjacent noncancerous tissues (Broadbent et al., 2008). NEAT1 is overexpressed in prostate cancer cells (Pasmant et al., 2011). The identification of lncRNA-disease associations (LDAs) helps us to further understand the biological processes and the molecular mechanisms of various complex diseases. However, the number of known and experimentally validated LDAs is very small. Thus, it is important to identify potential LDAs. Determining LDAs through *in vivo* experiments is costly and time-consuming, therefore, it is necessary to design efficient computational approaches for identifying potential LDAs (Meng et al., 2021; Peng et al., 2022d). Computational LDA prediction methods are categorized as biological network-based methods and machine learning-based methods.

Biological network-based methods use network algorithms for association prediction (Liu et al., 2023a). This type of method first constructs heterogeneous networks of lncRNAs and diseases and then identifies LDAs via matrix decomposition, random walk, and so on. To predict potential LDAs, LRWRHLDA combined Laplace normalized random walk with restart (Wang et al., 2022), LDGRNMF used graph regularized nonnegative matrix factorization (Wang et al., 2021), DSCMF developed a dual sparse collaborative matrix factorization approach (Liu et al., 2021a), RWSF-BLP added random walk-based multi-similarity fusion to bidirectional label propagation (Xie et al., 2021), HBRWRLDA utilized bi-random walk on hypergraphs (Xie et al., 2022), and MHRWRLDA exploited a random walk model with restart through multiplex and heterogeneous networks (Yao et al., 2021).

With the fast advance of RNA sequencing technologies, artificial intelligence has obtained wide applications in biomedical data analysis (Peng et al., 2023a; Peng et al., 2023b; Xu et al., 2023). Notably, artificial intelligence technologies, especially machine learning methods, have been widely applied to predict miRNA-disease associations (Liu et al., 2022) and circRNA-disease associations (Liu et al., 2023b). To find new LDAs, HGATLDA developed a novel heterogeneous graph attention network model (Zhao et al., 2022), DeepMNE extracted multi-omics data and designed a deep multi-network embedding model (Ma, 2022), iLncDA-LTR is a rank-based method (Wu et al., 2022), MAGCNSE utilized a graph convolutional network (Liang et al., 2022), LDAformer extracted topological features and used a transformer encoder for LDA classification (Zhou et al., 2022), BiGAN explored a bidirectional generative adversarial network (Yang et al., 2021), and SVDNVLDA extracted linear and non-linear features and used an XGBoost for LDA prediction (Li et al., 2021).

Computational methods have found many potential LDAs, however, network-based methods were more likely to favor well-investigated lncRNAs or diseases and can not predict LDAs for new lncRNAs or new diseases. Machine learning-based methods failed to effectively integrate different kernels from multiple data sources. Thus, in this study, we developed a machine learning-based method named LDAenDL to detect potential lncRNA biomarkers for lung cancer and neuroblastoma based on an ensemble of a deep neural network and LightGBM.

## 2 Materials and methods

**FIGURE 1:**
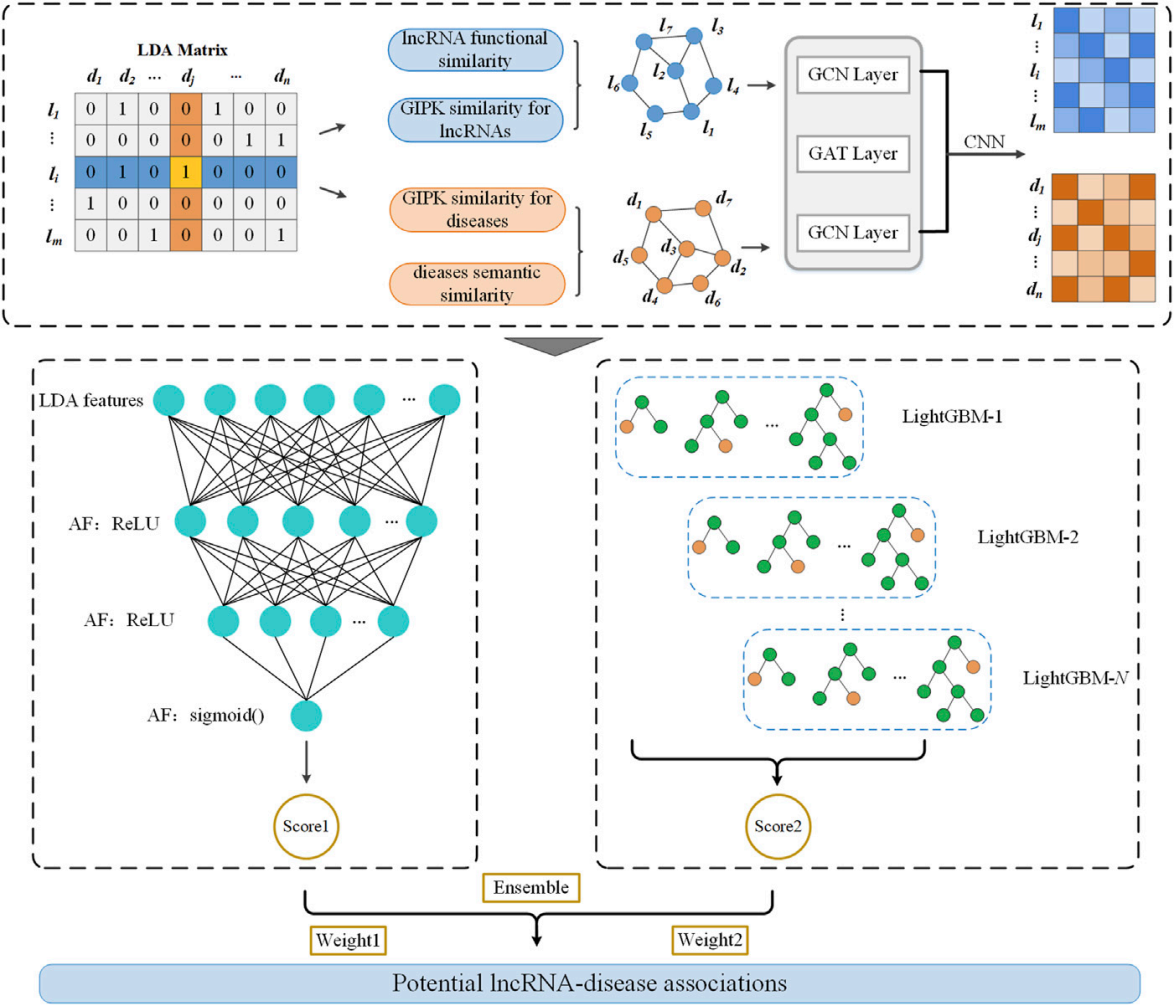
The pipeline of LDAenDL.

As shown in Figure 1, LDAenDL first computes the Gaussian kernel similarity and functional similarity of lncRNAs and the Gaussian kernel similarity and semantic similarity of diseases to obtain their similar networks. Next, LDAenDL combines a graph convolutional network (GCN) (Kipf and Welling, 2016), graph attention network (GAT) (Velickovic et al., 2017), and convolutional neural network (Gu et al., 2018) to learn the biological features of lncRNAs and diseases based on their similarity networks. Third, these features are concatenated and fed to an ensemble model composed of a deep neural network (DNN) and LightGBM to find new LDAs. Finally, LDAenDL was applied to identify possible lncRNA biomarkers associated with lung cancer and neuroblastoma.

### 2.1 Data preparation

We used two human LDA datasets that were provided by Chen et al. (2012) and Cui et al. (2018). Dataset 1 contains 605 LDAs between 157 diseases and 82 lncRNAs. Dataset 2 contains 1,529 LDAs between 190 diseases and 89 lncRNAs. An LDA network can be denoted as $Y\in R^{n\times m}$ where $y_{ij}=1$ if lncRNA $l_{i}$ interacts with disease $d_{j}$ , otherwise, it equals 0.

### 2.2 Similarity computation

Inspired by the LDA-DLPU method (Peng et al., 2022a), we computed the Gaussian kernel similarity and functional similarity of lncRNAs and the Gaussian kernel similarity and semantic similarity of diseases. Based on the computed lncRNA similarity and disease similarity matrices, we learned the features of lncRNAs and diseases by combining a GCN, GAT, and CNN.

### 2.3 Feature learning

Dai et al. (2022) designed a hybrid graph representation learning model (GraphCDA) to represent the features of circRNAs and diseases and obtained better circRNA-disease association prediction performance. Inspired by GraphCDA proposed by Dai et al. (2022), we exploit a GraphCDA-based LDA feature learning model.

#### 2.3.1 Graph convolutional network

A GCN was applied to obtain the feature representations of lncRNAs and diseases based on their similarity networks. For a GCN G, it is denoted as an adjacency matrix $S\in R^{N\times N}$ with N nodes where each node can be described as an F -dimensional vector. And GCN outputs node representation matrix $H^{new}$ in Eqs 1, 2:

$$H^{new}=GCN\left(S,H\right)$$ (1)

$$GCN\left(S,H\right)=\sigma \left(A^{-\frac{1}{2}}S^{\prime }A^{-\frac{1}{2}}HQ\right)$$ (2)

where $S^{\prime }=I+S$ , $A=\sum_{j}S_{i,j}^{\prime }$ and $Q\in R^{F\times F}$ denote degree matrix and trainable weight matrix, and σ(·) denotes a ReLU activation function.

#### 2.3.2 Graph attention network

A GAT (Veličković et al., 2017) uses multi-head attention to set weights for all adjacent nodes based on their importance. LDAenDL introduces a GAT layer between two GCN layers to help the GCN to extract high-level features of lncRNAs and diseases.

For the GCN G, a GAT layer outputs node representations $H^{new}$ in Eq. 3:

$$H^{new}=GAT\left(S,H\right)$$ (3)

For K attention mechanisms in multi-head attention and its weight matrix $W_{k}$ , let $\vec{H_{i}}$ denote the input feature vector of the i -th lncRNA, its feature representation ${\vec{H}}_{i}^{new}$ in $H^{new}$ can be denoted as Eq. 4:

$${\vec{H}}_{i}^{new}=\sigma \left(\frac{1}{K}\sum_{k=1}^{K}\sum_{j\neq i}^{n}\phi_{ij}^{k}W_{k}{\vec{H}}_{i}\right)$$ (4)

where $\phi_{it}^{k}$ denotes the k -th attention coefficients between two lncRNA nodes i and t :

$$\phi_{ij}^{k}=\frac{\exp\left(f\left(a_{k}^{T}\left[W_{k}{\vec{H}}_{i}∥W_{k}{\vec{H}}_{j}∥B_{k}S_{ij}\right]\right)\right)}{\sum_{t\neq i}\exp\left(f\left(a_{k}^{T}\left[W_{k}{\vec{H}}_{i}∥W_{k}{\vec{H}}_{t}∥B_{k}S_{it}\right]\right)\right)}$$ (5)

where || denotes a concatenation operation, f denotes the LeaklyReLU activation function, $a_{k}\in R^{2F+1}$ denotes a weight vector related to the k -th attention mechanism, and $B_{k}$ denotes the weight of an edge $S_{ij}$ .

#### 2.3.3 Feature representation of lncRNAs and diseases

For a lncRNA similarity network $G_{c}$ , its adjacency matrix C , and node feature matrix $H_{c}^{\left(0\right)}\in R^{N_{c}\times F_{c}}$ , we alternately use GCN and GAT layers to obtain the graph feature representation of lncRNAs at different levels in Eq. 6:

$$\left\{\begin{array}{c} H_{c}^{\left(1\right)}=GCN\left(C,H_{c}^{\left(0\right)}\right)\\ H_{c}^{\left(2\right)}=GAT\left(C,H_{c}^{\left(1\right)}\right)\\ H_{c}^{\left(3\right)}=GCN\left(C,H_{c}^{\left(2\right)}\right) \end{array}\right.$$ (6)

Thus, a 1D CNN is used to produce the lncRNA feature representation matrix $X_{c}$ by combining the output features $H_{c}^{\left(1\right)}$ and $H_{c}^{\left(3\right)}$ in the different GCN layers.

Similarly, the graph feature representations of diseases at different levels are denoted by Eq. 7:

$$\left\{\begin{array}{c} H_{d}^{\left(1\right)}=GCN\left(D,H_{d}^{\left(0\right)}\right)\\ H_{d}^{\left(2\right)}=GAT\left(D,H_{d}^{\left(1\right)}\right)\\ H_{d}^{\left(3\right)}=GCN\left(D,H_{d}^{\left(2\right)}\right) \end{array}\right.$$ (7)

A 1D CNN is used to produce the disease feature representation matrix $X_{c}$ by combining the output features $H_{d}^{\left(1\right)}$ and $H_{d}^{\left(3\right)}$ in the different GCN layers.

#### 2.3.4 Preference matrix construction

The preference matrix U that describes all lncRNA-disease pairs can be represented as Eq. 8 based on $X_{c}$ and $X_{d}$ :

$$U={X_{c}}^{T}X_{d}$$ (8)

We used binary cross-entropy as the activation function to evaluate the difference between the preference matrix U and the known adjacency matrix R . By minimizing the loss function on two LDA datasets, the feature representation matrices $X_{c}$ and $X_{d}$ of lncRNAs and diseases are learned.

### 2.4 LDA prediction

#### 2.4.1 DNN

We built a DNN to predict new LDAs based on known LDAs and the learned LDA features. The DNN contains an input layer, an output layer, and multiple hidden layers. In the input layer, there are F neurons that are the same as the number of LDA features.

Given an LDA sample x , the input layer with k inputs is represented by Eq. 9:

$$\left.{x=[x}_{1},x_{2},\ldots x_{k}\right]$$ (9)

where $x_{i}$ denotes the i -th feature in a sample x .

The hidden layer is represented by Eq. 10:

$$h_{j}=\sum_{i=1}^{k}\;w_{i}x_{i}+b_{j}$$ (10)

where $w_{i}$ and $b_{j}$ denote the weight of $x_{i}$ and the bias in the j -th hidden layer, respectively.

The output in the j -th hidden layer is denoted by Eq. 11:

$$h=f\left(h_{j}\right)$$ (11)

where f denotes a ReLU activation function. Finally, the output layer with the sigmoid function outputs the LDA prediction results in Eq. 12:

$$\sigma \left(h\right)=\frac{1}{1+e^{-h}}$$ (12)

#### 2.4.2 LightGBM

In this section, we built a LightGBM (Ke et al., 2017) to identify new LDAs. For a training set $X={\left\{\left(x_{i},y_{i}\right)\right\}}_{i=1}^{n}$ with n lncRNA-disease pair, LightGBM intends to build an approximation of $\hat{f}$ to a certain function $f\left(x\right)$ by minimizing the expected value of loss function $L\left(y,f\left(x\right)\right)$ by Eq. 13:

$$\hat{f}=\arg\min_{f}\;E_{x,y}\left[L\left(y,f\left(x\right)\right)\right]$$ (13)

LightGBM integrates T regression trees $\sum_{t=1}^{T}\;f_{t}\left(X\right)$ to approximate the final model by Eq. 14:

$$f_{T}\left(X\right)=\sum_{t=1}^{T}f_{t}\left(X\right)$$ (14)

The regression trees are expressed as $w_{q\left(x\right)},q\in \left\{1,2,\ldots ,J\right\}$ , where J , q , and w denote the number of leaves, the decision rules of the tree, and the sample weight of leaf nodes, respectively.

At step t , LightGBM is trained in an additive form:

$$\Gamma_{t}=\sum_{i=1}^{n}L\left(y_{i},F_{t-1}\left(x_{i}\right)+f_{t}\left(x_{i}\right)\right)$$ (15)

The objective function (15) is rapidly approximated with Newton’s method (Sun et al., 2020).

To solve the objective function of LightGBM, we removed the constant term for simplicity, and model (15) can be represented as Eq. 16:

$$\Gamma_{t}≅\sum_{i=1}^{n}\left(g_{i}f_{t}\left(x_{i}\right)+\frac{1}{2}h_{i}f_{t}^{2}\left(x_{i}\right)\right)$$ (16)

where $g_{i}$ and $h_{i}$ are the first-order and second-order gradients related to the loss function. Given the sample set $I_{j}$ related to leaf j , Eq. 16 is transformed to Eq. 17:

$$\Gamma_{t}=\sum_{j=1}^{J}\left(\left(\sum_{i\in I_{j}}g_{i}\right)w_{j}+\frac{1}{2}\left(\sum_{i\in I_{j}}h_{i}+\lambda \right)w_{j}^{2}\right)$$ (17)

Given a certain tree structure $q\left(x\right)$ , for each leaf node $w_{j}^{*}$ , its optimal leaf weight and the extreme value of $\Gamma_{k}$ could be computed by Eq. 18:

$$\begin{array}{c} w_{j}^{*}=-\frac{\sum_{i\in I_{j}}g_{i}}{\sum_{i\in I_{j}}h_{i}+\lambda }\\ \Gamma_{T}^{*}=-\frac{1}{2}\sum_{j=1}^{J}\frac{{\left(\sum_{i\in I_{j}}g_{i}\right)}^{2}}{\sum_{i\in I_{j}}h_{i}+\lambda } \end{array}$$ (18)

where $\Gamma_{T}^{*}$ is a scoring function used to evaluate the quality of a tree structure q . Finally, Model (15) can be denoted as:

$$G=\frac{1}{2}\left(\frac{{\left(\sum_{i\in I_{L}}g_{i}\right)}^{2}}{\sum_{i\in I_{L}}h_{i}+\lambda }+\frac{{\left(\sum_{i\in I_{R}}g_{i}\right)}^{2}}{\sum_{i\in I_{R}}h_{i}+\lambda }-\frac{{\left(\sum_{i\in I}g_{i}\right)}^{2}}{\sum_{i\in I}h_{i}+\lambda }\right)$$ (19)

where $I_{L}$ and $I_{R}$ denote the example sets in the left and right subtrees of q , respectively.

#### 2.4.3 Ensemble learning

Through the solution of models (12) and (15), we can identify potential LDAs based on a DNN and LightGBM. Ensemble learning has better prediction accuracy than a single model. To further improve LDA prediction accuracy, we combined a DNN and LightGBM and developed an ensemble model for LDA identification through soft voting in Eq. 16:

$$Score=\alpha C_{DNN}+\beta C_{LightGBM}$$ (20)

where $C_{DNN}$ and $C_{LightGBM}$ denote LDA prediction results from the DNN and LightGBM, respectively. α and β are their weights with values of 0.4 and 0.6, respectively. In particular, a lncRNA–disease pair is taken as an LDA if its association probability is greater than 0.5; otherwise, the pair is taken as a negative LDA.

## 3 Results

### 3.1 Evaluation metrics

In this article, we compared our proposed LDAenDL method with four LDA prediction methods, SDLDA, LDNFSGB, IPCAF, and LDASR. Precision, recall, accuracy, F1-score, AUC, and AUPR were used to compare the performance of LDAenDL with the four methods. The six metrics have been defined by Peng et al. (2022b) (Shen et al., 2022).

### 3.2 Comparison of LDAenDL with the other four methods

To implement the performance evaluation, inspired by the three cross-validations proposed by Zhou et al. (2021), we conducted cross-validations on lncRNAs (CV1), diseases (CV2), and lncRNA-disease pairs (CV3). Tables 1–3 give the precision, recall, accuracy, F1-score, AUC, and AUPR under CV1, CV2, and CV3 on two LDA datasets. In Tables 1–6, the bold font in each row denotes the best performance.

**TABLE 1:** Comparison of LDAenDL with the other four methods under CV1.

		SDLDA	LDNFSGB	IPCARF	LDASR	LDAenDL
Precision	Dataset 1	0.8514 ± 0.0509	0.7004 ± 0.0639	0.4878 ± 0.1309	0.6726 ± 0.1200	**0.8764 ± 0.0493**
	Dataset 2	**0.9399 ± 0.0154**	0.8552 ± 0.0393	0.6615 ± 0.0966	0.8405 ± 0.0300	0.9391 ± 0.0290
Recall	Dataset 1	0.6521 ± 0.0732	0.6092 ± 0.0790	0.5721 ± 0.1580	0.5129 ± 0.0946	**0.7019 ± 0.0639**
	Dataset 2	0.8239 ± 0.0437	0.8021 ± 0.0498	0.6434 ± 0.1545	0.7358 ± 0.0562	**0.8304 ± 0.0523**
Accuracy	Dataset 1	0.7799 ± 0.0341	0.6769 ± 0.0423	0.4906 ± 0.0951	0.6417 ± 0.0597	**0.7996 ± 0.0312**
	Dataset 2	0.8857 ± 0.0283	0.8323 ± 0.0230	0.6526 ± 0.0775	0.7972 ± 0.0268	**0.8879 ± 0.0289**
F1-score	Dataset 1	0.7365 ± 0.0563	0.6462 ± 0.0451	0.5125 ± 0.1100	0.5668 ± 0.0536	**0.7768 ± 0.0399**
	Dataset 2	0.8775 ± 0.0278	0.8260 ± 0.0230	0.6401 ± 0.1017	0.7827 ± 0.0260	**0.8804 ± 0.0334**
AUC	Dataset 1	0.8023 ± 0.0477	0.7346 ± 0.0465	0.5096 ± 0.1432	0.7057 ± 0.0420	**0.8701 ± 0.0339**
	Dataset 2	0.9366 ± 0.0195	0.8839 ± 0.0270	0.7104 ± 0.0997	0.8641 ± 0.0256	**0.9490 ± 0.0220**
AUPR	Dataset 1	0.8461 ± 0.0553	0.7239 ± 0.0626	0.5336 ± 0.1423	0.6775 ± 0.0971	**0.8903 ± 0.0273**
	Dataset 2	0.9533 ± 0.0129	0.8832 ± 0.0307	0.7128 ± 0.1012	0.8671 ± 0.0252	**0.9582 ± 0.0167**

The bold value denotes the best performance.

**TABLE 2:** Comparison of LDAenDL with the other four methods under CV2.

		SDLDA	LDNFSGB	IPCARF	LDASR	LDAenDL
Precision	Dataset 1	0.8854 ± 0.0377	0.7548 ± 0.0639	0.5583 ± 0.0910	0.7462 ± 0.0613	**0.9135 ± 0.0317**
	Dataset 2	0.9232 ± 0.0331	0.8005 ± 0.0625	0.5557 ± 0.1473	0.7625 ± 0.0749	**0.9528 ± 0.0225**
Recall	Dataset 1	**0.7182 ± 0.0694**	0.7309 ± 0.0646	0.7538 ± 0.1067	0.6431 ± 0.0757	0.6649 ± 0.0814
	Dataset 2	**0.8579 ± 0.0655**	0.6936 ± 0.0794	0.5279 ± 0.1969	0.5758 ± 0.0894	0.4616 ± 0.1702
Accuracy	Dataset 1	**0.8187 ± 0.0282**	0.7552 ± 0.0291	0.5766 ± 0.0740	0.7165 ± 0.0339	0.8005 ± 0.0381
	Dataset 2	**0.9043 ± 0.0174**	0.7670 ± 0.0432	0.5593 ± 0.1159	0.7010 ± 0.0463	0.7196 ± 0.0821
F1-score	Dataset 1	**0.7917 ± 0.0519**	0.7407 ± 0.0526	0.6339 ± 0.0715	0.6873 ± 0.0512	0.7664 ± 0.0593
	Dataset 2	**0.8886 ± 0.0475**	0.7402 ± 0.0577	0.5190 ± 0.1434	0.6485 ± 0.0555	0.6032 ± 0.1612
AUC	Dataset 1	0.8788 ± 0.0274	0.8329 ± 0.0273	0.6402 ± 0.1004	0.7951 ± 0.0317	**0.8953 ± 0.0284**
	Dataset 2	**0.9559 ± 0.0160**	0.8603 ± 0.0363	0.5992 ± 0.1601	0.8045 ± 0.0362	0.9157 ± 0.0420
AUPR	Dataset 1	0.8934 ± 0.0387	0.8163 ± 0.0537	0.6355 ± 0.1217	0.7914 ± 0.0542	**0.9061 ± 0.0254**
	Dataset 2	**0.9561 ± 0.0354**	0.8292 ± 0.0680	0.6040 ± 0.1476	0.7630 ± 0.0717	0.9122 ± 0.0436

The bold value denotes the best performance.

**TABLE 3:** Comparison of LDAenDL with the other four methods under CV3.

		SDLDA	LDNFSGB	IPCARF	LDASR	LDAenDL
Precision	Dataset 1	**0.8782 ± 0.0306**	0.7782 ± 0.0270	0.7069 ± 0.0478	0.7695 ± 0.0393	0.8637 ± 0.0312
	Dataset 2	0.9178 ± 0.0154	0.8548 ± 0.0156	0.7693 ± 0.0850	0.8553 ± 0.0189	**0.9351 ± 0.0157**
Recall	Dataset 1	0.7256 ± 0.0376	0.8169 ± 0.0408	0.6155 ± 0.0652	0.6836 ± 0.0342	**0.8234 ± 0.0314**
	Dataset 2	0.8824 ± 0.0198	0.8818 ± 0.0204	0.5034 ± 0.1469	0.8204 ± 0.0238	**0.8999 ± 0.0179**
Accuracy	Dataset 1	0.8120 ± 0.0216	0.7916 ± 0.0256	0.6793 ± 0.0403	0.7385 ± 0.0283	**0.8462 ± 0.0229**
	Dataset 2	0.9015 ± 0.0114	0.8658 ± 0.0127	0.6793 ± 0.0753	0.8405 ± 0.0129	**0.9186 ± 0.0126**
F1-score	Dataset 1	0.7939 ± 0.0260	0.7965 ± 0.0262	0.6563 ± 0.0492	0.7233 ± 0.0289	**0.8426 ± 0.0232**
	Dataset 2	0.8996 ± 0.0119	0.8679 ± 0.0129	0.5995 ± 0.1312	0.8371 ± 0.0137	**0.9171 ± 0.0130**
AUC	Dataset 1	0.8774 ± 0.0200	0.8578 ± 0.0234	0.7384 ± 0.0466	0.8133 ± 0.0218	**0.9110 ± 0.0197**
	Dataset 2	0.9560 ± 0.0081	0.9346 ± 0.0074	0.7680 ± 0.0882	0.9143 ± 0.0112	**0.9708 ± 0.0062**
AUPR	Dataset 1	0.8952 ± 0.0177	0.8489 ± 0.0289	0.7409 ± 0.0515	0.8131 ± 0.0277	**0.9166 ± 0.0203**
	Dataset 2	0.9639 ± 0.0063	0.9273 ± 0.0098	0.7689 ± 0.0924	0.9100 ± 0.0136	**0.9743 ± 0.0058**

The bold value denotes the best performance.

**TABLE 4:** Comparison of LDAenDL with individual models under CV1.

		DNN	LightGBM	LDAenDL
Precision	Dataset 1	**0.8772 ± 0.0461**	0.8569 ± 0.0511	0.8764 ± 0.0493
	Dataset 2	0.9149 ± 0.0375	0.9386 ± 0.0278	**0.9391 ± 0.0290**
Recall	Dataset 1	0.6851 ± 0.0694	0.7106 ± 0.0714	**0.7019 ± 0.0639**
	Dataset 2	**0.8337 ± 0.0510**	0.8278 ± 0.0533	0.8304 ± 0.0523
Accuracy	Dataset 1	0.7930 ± 0.0317	0.7939 ± 0.0340	**0.7996 ± 0.0312**
	Dataset 2	0.8772 ± 0.0288	0.8865 ± 0.0295	**0.8879 ± 0.0289**
F1-score	Dataset 1	0.7664 ± 0.0429	0.7737 ± 0.0446	**0.7768 ± 0.0399**
	Dataset 2	0.8711 ± 0.0321	0.8786 ± 0.0344	**0.8804 ± 0.0334**
AUC	Dataset 1	**0.8712 ± 0.0373**	0.8622 ± 0.0340	0.8701 ± 0.0339
	Dataset 2	0.9308 ± 0.0209	**0.9497 ± 0.0227**	0.9490 ± 0.0220
AUPR	Dataset 1	0.8842 ± 0.0327	0.8822 ± 0.0284	**0.8903 ± 0.0273**
	Dataset 2	0.9449 ± 0.0190	**0.9586 ± 0.0171**	0.9582 ± 0.0167

The bold value denotes the best performance.

**TABLE 5:** Comparison of LDAenDL with individual models under CV2.

		DNN	LightGBM	LDAenDL
Precision	Dataset 1	0.9049 ± 0.0383	0.8927 ± 0.0309	**0.9135 ± 0.0317**
	Dataset 2	0.9274 ± 0.0412	0.9439 ± 0.0283	**0.9528 ± 0.0225**
Recall	Dataset 1	0.6182 ± 0.1006	**0.6873 ± 0.0734**	0.6649 ± 0.0814
	Dataset 2	0.3426 ± 0.1457	**0.5370 ± 0.1739**	0.4616 ± 0.1702
Accuracy	Dataset 1	0.7759 ± 0.0453	**0.8017 ± 0.0336**	0.8005 ± 0.0381
	Dataset 2	0.6580 ± 0.0689	**0.7533 ± 0.0842**	0.7196 ± 0.0821
F1-score	Dataset 1	0.7289 ± 0.0794	**0.7740 ± 0.0493**	0.7664 ± 0.0593
	Dataset 2	0.4835 ± 0.1531	**0.6678 ± 0.1537**	0.6032 ± 0.1612
AUC	Dataset 1	0.8853 ± 0.0374	0.8869 ± 0.0281	**0.8953 ± 0.0284**
	Dataset 2	0.8412 ± 0.0512	**0.9164 ± 0.0441**	0.9157 ± 0.0420
AUPR	Dataset 1	0.8882 ± 0.0368	0.8981 ± 0.0257	**0.9061 ± 0.0254**
	Dataset 2	0.8416 ± 0.0530	**0.9150 ± 0.0466**	0.9122 ± 0.0436

The bold value denotes the best performance.

**TABLE 6:** Comparison of LDAenDL with individual models under CV3.

		DNN	LightGBM	LDAenDL
Precision	Dataset 1	0.8561 ± 0.0357	0.8477 ± 0.0320	**0.8637 ± 0.0312**
	Dataset 2	0.9214 ± 0.0171	0.9322 ± 0.0157	**0.9351 ± 0.0157**
Recall	Dataset 1	**0.8241 ± 0.0373**	0.8110 ± 0.0381	0.8234 ± 0.0314
	Dataset 2	0.8983 ± 0.0204	0.8936 ± 0.0176	**0.8999 ± 0.0179**
Accuracy	Dataset 1	0.8419 ± 0.0244	0.8322 ± 0.0265	**0.8462 ± 0.0229**
	Dataset 2	0.9106 ± 0.0130	0.9142 ± 0.0122	**0.9186 ± 0.0126**
F1-score	Dataset 1	0.8389 ± 0.0247	0.8284 ± 0.0277	**0.8426 ± 0.0232**
	Dataset 2	0.9095 ± 0.0134	0.9124 ± 0.0126	**0.9171 ± 0.0130**
AUC	Dataset 1	0.9076 ± 0.0225	0.9015 ± 0.0204	**0.9110 ± 0.0197**
	Dataset 2	0.9562 ± 0.0107	0.9692 ± 0.0064	**0.9708 ± 0.0062**
AUPR	Dataset 1	0.9067 ± 0.0244	0.9082 ± 0.0215	**0.9166 ± 0.0203**
	Dataset 2	0.9611 ± 0.0102	0.9728 ± 0.0061	**0.9743 ± 0.0058**

The bold value denotes the best performance.

Under CV1, LDAenDL randomly took 80% of lncRNAs as training samples, and the rest were taken as test samples to investigate the LDA prediction ability for new lncRNAs. The results from Table 1 show that our proposed LDAenDL approach obtained the best precision, recall, accuracy, F1-score, AUC, and AUPR on two datasets under CV1 except that it computed slightly lower precision on Dataset 2 (0.9391 vs. 0.9399). It computed the highest AUPRs of 0.8903 and 0.9582, and far exceeded the AUPR values computed by SDLDA (i.e., 0.8461 and 0.9533).

**FIGURE 2:**
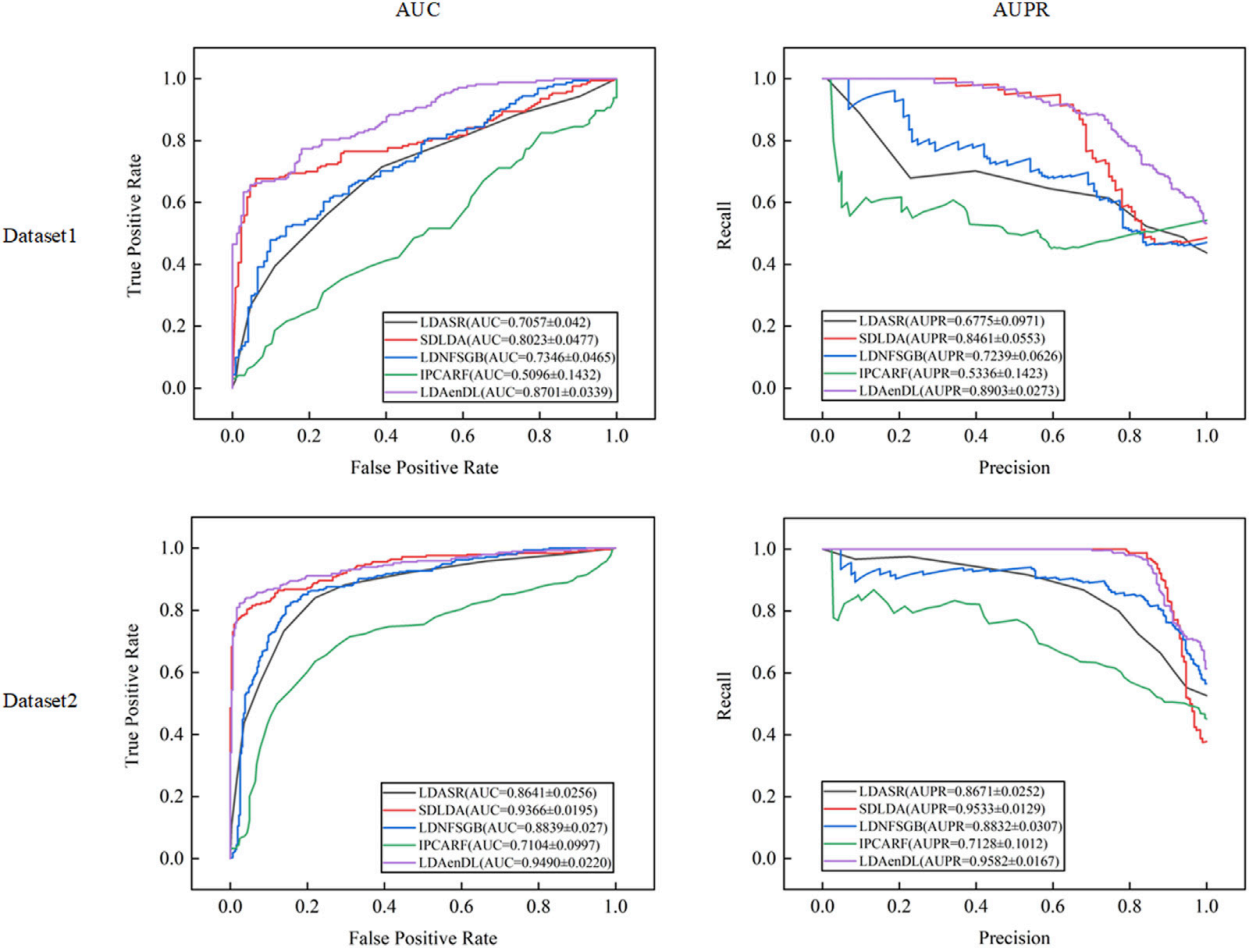
The AUC and AUPR values of five LDA prediction methods under CV1.

Figure 2 shows the AUC and AUPR values computed by LDAenDL and the other four methods on two datasets under CV1. The results demonstrated that LDAenDL can discover possible diseases associated with a new lncRNA.

Under CV2, LDAenDL randomly took 80% of diseases as training samples, and the rest were taken as test samples to investigate the LDA prediction ability for new diseases. The results from Table 2 show that our proposed LDAenDL approach obtained better precision, AUC, and AUPR on two datasets under CV2. However, SDLDA computed higher recall, accuracy, and F1-score than LDAenDL, which may be caused by smaller disease samples.

**FIGURE 3:**
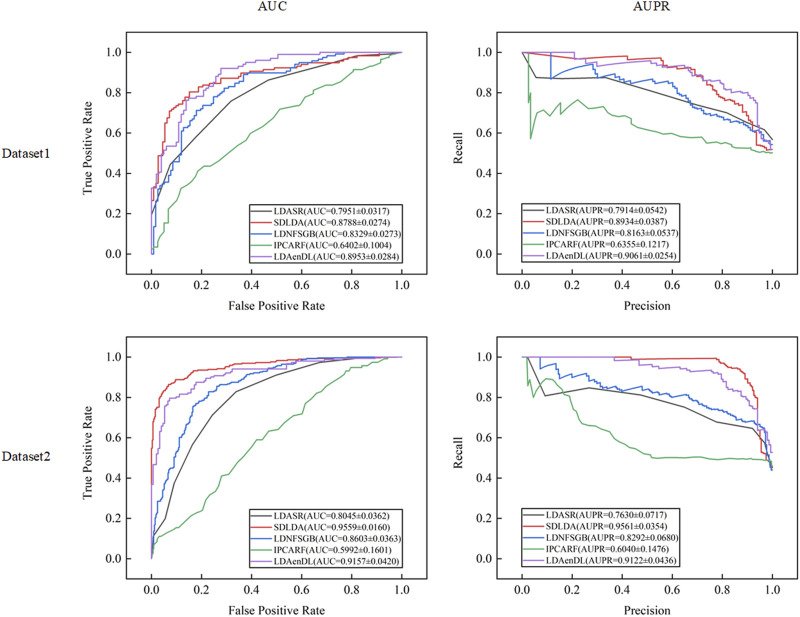
The AUC and AUPR values of five LDA prediction methods under CV2.

Figure 3 shows the AUC and AUPR values computed by LDAenDL and the other four methods on two datasets under CV2. The results show that LDAenDL can be applied to screen possible lncRNAs associated with a new disease.

Under CV3, LDAenDL randomly took 80% of lncRNA-disease pairs as training samples, and the rest were taken as test samples to investigate the LDA prediction ability. The results from Table 3 show that our proposed LDAenDL approach obtained the best precision, recall, accuracy, F1-score, AUC, and AUPR on two datasets under CV3. It computed the highest AUCs of 0.9110 and 0.9708 and far exceeded those computed by SDLDA (i.e., 0.8774 and 0.9560). Furthermore, our LDAenDL approach computed the highest AUPRs of 0.9166 and 0.9743 and far exceeded those computed by SDLDA (i.e., 0.8952, and 0.9639).

**FIGURE 4:**
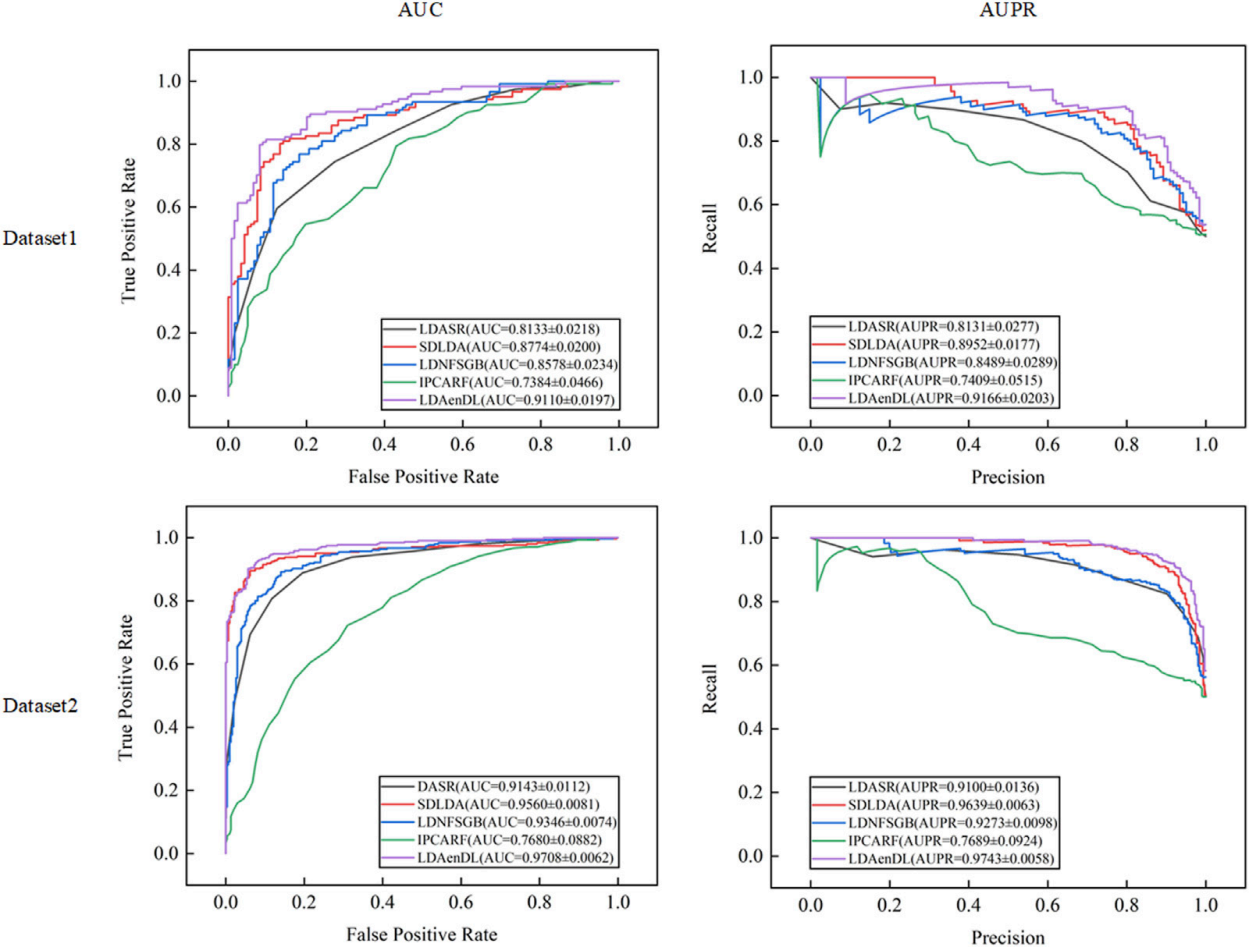
The AUC and AUPR values of five LDA prediction methods under CV3.

Figure 4 shows the AUC and AUPR values computed by LDAenDL and the other four methods on two datasets under CV3. The results demonstrated that LDAenDL could find potential LDAs based on known LDAs.

### 3.3 Comparison of LDAenDL with individual models

To measure the effect of the ensemble algorithm on LDA prediction performance, we compared LDAenDL with two individual models, DNN, and LightGBM. Tables 4–6 show the precision, recall, accuracy, F1-score, AUC, and AUPR of the DNN, LightGBM, and LDAenDL under CV1, CV2, and CV3, respectively.

Under CV1, as shown in Table 4, LDAenDL outperformed the DNN and LightGBM on two LDA datasets for the majority of conditions. LDAenDL computed the best accuracy and F1-score on the two datasets. Although LDAenDL computed slightly lower AUC value than the DNN on dataset 1, and still slightly lower AUC than LightGBM on dataset 2, their differences were very small. For example, the DNN computed an AUC of 0.8712 while LDAenDL computed 0.8701 on dataset 1, and the DNN calculated an AUC of 0.9497 while LDAenDL calculated 0.9490 on dataset 2. LDAenDL obtained the best AUPR on dataset 1, and LightGBM obtained an AUPR of 0.9586 while LDAenDL obtained an AUPR of 0.9582.

Under CV2, as shown in Table 5, LDAenDL outperformed the DNN under all conditions on two LDA datasets. Recall, accuracy, and F1-score computed by LightGBM were slightly better than LDAenDL on the two datasets. But it calculated the best AUC and AUPR on dataset 1.

Under CV3, as shown in Table 6, LDAenDL computed the highest precision, recall, accuracy, F1-score, AUC, and AUPR on the two LDA datasets except that it computed a slightly lower recall on dataset 1. The results demonstrate that LDAenDL is appropriate to predict possible LDAs from unknown lncRNA-disease pairs.

### 3.4 Case study

#### 3.4.1 Identifying possible lncRNA biomarkers for lung cancer

Lung cancer is one of the most prevalent causes of mortality globally. It mainly contains small cell lung cancer and non-small cell lung cancer. Targeted drug therapy is its one therapeutic option (Lahiri et al., 2023). We used the proposed LDAenDL method to predict possible lncRNA biomarkers for lung cancer. Table 7 shows the predicted top 20 lncRNA biomarkers for lung cancer. The 20 lncRNA biomarkers associated with lung cancer have no known association information with lung cancer in the two datasets.

**TABLE 7:** The predicted top 20 lncRNA biomarkers for lung cancer in each of the two datasets.

Dataset 1			Dataset 2		
Rank	lncRNA	Evidence	Rank	lncRNA	Evidence
1	TUG1	27485439, 31532756	1	TUG1	27485439, 31532756
2	CRNDE	28550688, 30982057	2	DLEU2	31721438
3	DANCR	30535487, 32196604	3	WT1-AS	32349718
4	MIAT	29795987	4	CRNDE	28550688, 30982057
5	NPTN-IT1	27896272, 29416684	5	DANCR	30535487, 32196604
6	HNF1A-AS1	25863539	6	SNHG11	32239719
7	LINC00032	Unconfirmed	7	IFNG-AS1	Unconfirmed
8	WT1-AS	32349718	8	HULC	30575912
9	CBR3-AS1	32945466	9	XIST	29812958
10	HULC	30575912	10	PCA3	Unconfirmed
11	CCDC26	Unconfirmed	11	SRA1	Unconfirmed
12	SNHG3	31602642	12	HAR1A	Unconfirmed
13	PVT1	27904703	13	DSCAM-AS1	32280246
14	BCAR4	28537678	14	NPTN-IT1	27896272, 29416684
15	PTENP1	32698750	15	TCL6	Unconfirmed
16	RMST	Unconfirmed	16	PTENP1	32698750
17	LSINCT5	20214974	17	PANDAR	28121347
18	MIR155HG	32432745	18	TDRG1	31742752
19	BOK-AS1	Unconfirmed	19	KCNQ1OT1	31486494
20	KCNQ1OT1	31486494	20	IGF2-AS	28471495

In dataset 1, LDAenDL predicted that CCDC26 could be associated with lung cancer. CCDC26 can enhance thyroid cancer malignant progression (Ma et al., 2021). It promotes imatinib resistance in human gastrointestinal stromal tumors (Yan et al., 2019). Its inhibition could increase the sensitivity of doxorubicin in MDR-CML cells (Liu et al., 2021b). In this study, we predicted that CCDC26 could be associated with lung cancer in dataset 1.

In dataset 2, LDAenDL predicted that IFNG-AS1 could be associated with lung cancer. IFNG-AS1 has been reported in long-lasting memory T cells (Castellucci et al., 2021). It can boost interferon gamma generation in human natural killer cells (Stein et al., 2019). We identified that IFNG-AS1 could be associated with lung cancer in Dataset 2.

**FIGURE 5:**
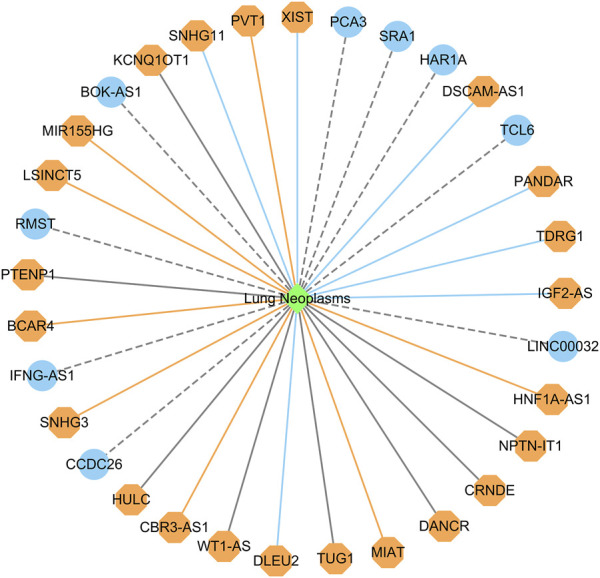
The top 20 predicted lncRNA biomarkers for lung cancer in each of the two datasets (The repeated lncRNAs in the two datasets have been removed). This figure was drawn using Cytoscape (Shannon et al., 2003).

Figure 5 shows the top 20 predicted lncRNAs associated with lung cancer in each of the two datasets. Yellow solid lines and blue solid lines denote lncRNA-lung cancer associations confirmed by the literatures among the predicted top 20 associations on datasets 1 and 2, respectively. Grey solid lines denote the predicted and co-occurring lncRNA-lung cancer associations that can be confirmed by the literatures in the two datasets, and grey dashed lines denote the predicted and unconfirmed lncRNA-lung cancer associations in the two datasets. The repeated lncRNAs in the two datasets have been removed.

#### 3.4.2 Identifying possible lncRNAs associated with PDL1 for lung cancer

Recent advances in lung cancer treatment have demonstrated significant responses in patients when they were treated with programmed death-1/programmed death-ligand 1 (PD-1/PD-L1) checkpoint blockade immunotherapies (Lahiri et al., 2023). To find possible lncRNAs associated with PDL1 for lung cancer, inspired by LPI-DLDN proposed by Peng et al. (2022a), we first downloaded the sequence of PDL1 from the UniProt database. Next, we extracted the biological features of PDL1 and depicted PDL1 as a 10,029-dimensional vector using BioTriangle. Finally, we used cosine similarity to compute the similarities between PDL1 and the other proteins in a lncRNA-protein interaction dataset (Li et al., 2015) and found the top 3 proteins with the highest interaction probabilities with PDL1. The results show that SNHG3 has a higher interaction probability with PDL1 and has been reported to be associated with lung cancer.

#### 3.4.3 Identifying possible lncRNA biomarkers for neuroblastoma

Neuroblastoma is the most frequent pediatric solid tumor and accounts for approximately 15% of childhood cancer-related mortality (Zafar et al., 2021). We used the proposed LDAenDL method to identify possible lncRNA biomarkers for neuroblastoma. Table 8 shows the top 20 predicted lncRNA biomarkers for neuroblastoma in each of the two datasets. The repeated lncRNAs in the two datasets have been removed.

**TABLE 8:** The top 20 predicted lncRNA biomarkers for neuroblastoma in each of the two datasets.

Dataset 1			Dataset 2		
Rank	lncRNA	Evidence	Rank	lncRNA	Evidence
1	HOTAIR	Unconfirmed	1	BDNF-AS	Unconfirmed
2	HNF1A-AS1	Unconfirmed	2	SNHG4	32614236
3	CDKN2B-AS1	Unconfirmed	3	BANCR	Unconfirmed
4	GAS5	28035057	4	HAR1A	Unconfirmed
5	CCAT1	Unconfirmed	5	HCP5	33189302
6	TUG1	Unconfirmed	6	TUG1	Unconfirmed
7	UCA1	Unconfirmed	7	HOTAIR	Unconfirmed
8	CRNDE	Unconfirmed	8	SRA1	Unconfirmed
9	WT1-AS	Unconfirmed	9	TERC	Unconfirmed
10	BANCR	Unconfirmed	10	SPRY4-IT1	Unconfirmed
11	WRAP53	Unconfirmed	11	KCNQ1OT1	31433907
12	SPRY4-IT1	Unconfirmed	12	IGF2-AS	30914706
13	CCAT2	33475889	13	PTENP1	Unconfirmed
14	CCDC26	Unconfirmed	14	CCAT1	Unconfirmed
15	PVT1	Unconfirmed	15	PCAT1	Unconfirmed
16	HULC	Unconfirmed	16	NPTN-IT1	Unconfirmed
17	CASC2	Unconfirmed	17	DGCR5	Unconfirmed
18	DANCR	34050113	18	HULC	Unconfirmed
19	KCNQ1OT1	31433907	19	BOK-AS1	Unconfirmed
20	7SK	Unconfirmed	20	BCYRN1	Unconfirmed

In dataset 1, we predicted that HOTAIR could be associated with neuroblastoma with the highest probability. HOTAIR is a novel oncogenic biomarker in human cancer (Rajagopal et al., 2020). Its knockdown can promote radiosensitivity in colorectal cancer (Liu et al., 2020). It also can enhance the carcinogenesis of gastric (Zhang et al., 2020). We identified that HOTAIR may be one biomarker of neuroblastoma in dataset 1.

In dataset 2, we predicted that BDNF-AS could be associated with neuroblastoma with the highest probability. PABPC1-induced stabilization of BDNF-AS helps the inhibition of malignant progression in glioblastoma cells (Su et al., 2020). It can regulate the miR-9-5p/BACE1 pathway that affects neurotoxicity in Alzheimer’s disease (Ding et al., 2022). We identified that BDNF-AS is a possible biomarker of neuroblastoma in dataset 2.

**FIGURE 6:**
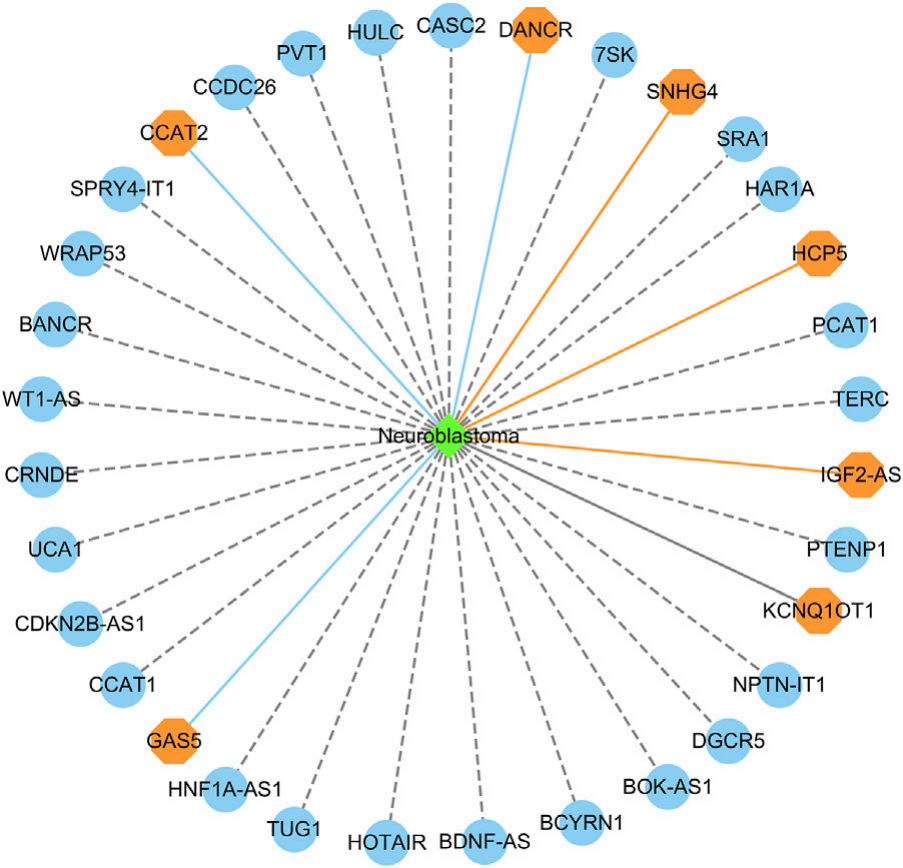
The top 20 predicted lncRNA biomarkers for neuroblastoma in each of the two datasets. (The repeated lncRNAs in the two datasets have been removed). This figure was drawn using Cytoscape (Shannon et al., 2003).

Figure 6 shows the top 20 predicted lncRNAs associated with neuroblastoma in each of the two datasets. Yellow solid lines and blue solid lines denote lncRNA-neuroblastoma associations confirmed by the literatures among the predicted top 20 associations on datasets 1 and 2, respectively. Grey solid lines denote the predicted and co-occurring lncRNA-neuroblastoma associations that can be confirmed by the literatures in the two datasets, and grey dashed lines denote the predicted and unconfirmed lncRNA-neuroblastoma associations in the two datasets. The repeated lncRNAs in the two datasets have been removed.

## 4 Conclusion

Lung cancer and neuroblastoma are two human diseases that severely affect the human body. Detecting new biomarkers for them contributes to their diagnosis and therapy. Experimental biomarker identification methods are costly and laborious. Thus, we developed a machine learning-based method named LDAenDL to predict possible lncRNA biomarkers for the two diseases based on an ensemble of a deep neural network and LightGBM. LDAenDL first computed lncRNA similarity and disease similarity and then combined a GCN, GAT, and CNN to learn the biological features of lncRNAs and diseases. Finally, these features were fed to a DNN and LightGBM to find new LDAs.

LDAenDL was compared with the other four classical LDA prediction methods (i.e., SDLDA, LDNFSGB, IPCAF, and LDASR). The results showed that LDAenDL computed the best AUCs and AUPRs under three cross-validations on two LDA datasets, demonstrating the optimal LDA prediction performance of LDAenDL. We further identified possible lncRNA biomarkers for lung cancer and neuroblastoma. The results demonstrated that CCDC26 and IFNG-AS1 may be new biomarkers for lung cancer, SNHG3 may be associated with PDL1 for lung cancer, and HOTAIR and BDNF-AS may be potential biomarkers for neuroblastoma.

In the future, we will combine data from multiple sources, for example, miRNA, circRNA, and drugs, to improve LDA identification performance. We will also design a new deep-learning model to efficiently extract the biological features of lncRNAs and diseases for LDA prediction. We hope that the proposed LDAenDL can help the development of targeted therapies for these two diseases.

## Data Availability

The original contributions presented in the study are included in the article/Supplementary Material, further inquiries can be directed to the corresponding authors.
